# Diverse Effects of Glutathione and UPF Peptides on Antioxidant Defense System in Human Erythroleukemia Cells K562

**DOI:** 10.1155/2012/124163

**Published:** 2012-02-15

**Authors:** Ceslava Kairane, Riina Mahlapuu, Kersti Ehrlich, Kalle Kilk, Mihkel Zilmer, Ursel Soomets

**Affiliations:** The Centre of Excellence of Translational Medicine, Department of Biochemistry, Faculty of Medicine, University of Tartu, Ravila Street 19, 50411 Tartu, Estonia

## Abstract

The main goal of the present paper was to examine the influence of the replacement of **γ**-Glu moiety to **α**-Glu in glutathione and in its antioxidative tetrapeptidic analogue UPF1 (Tyr(Me)-**γ**-Glu-Cys-Gly), resulting in **α**-GSH and UPF17 (Tyr(Me)-Glu-Cys-Gly), on the antioxidative defense system in K562 cells. UPF1 and GSH increased while UPF17 and **α**-GSH decreased the activity of CuZnSOD in K562 cells, at peptide concentration of 10 **μ**M by 42% and 38% or 35% and 24%, respectively. After three-hour incubation, UPF1 increased and UPF17 decreased the intracellular level of total GSH. Additionally, it was shown that UPF1 is not degraded by **γ**-glutamyltranspeptidase, which performs glutathione breakdown. These results indicate that effective antioxidative character of peptides does not depend only on the reactivity of the thiol group, but also of the other functional groups, and on the spatial structure of peptides.

## 1. Introduction

Glutathione (GSH) system is an attractive target for drug discovery because of its importance and versatility [1]. GSH (*γ*-L-Glu-L-Cys-Gly) is a prevalent low molecular weight thiol in eukaryotic cells and has antioxidative, detoxificative, and regulatory roles [2, 3]. Decrease of GSH level and shifted GSH redox status are related to several pathological states, including neurodegenerative, cardiovascular, pulmonary, and immune system diseases [4]. Exogenous administration of GSH to compensate the decrease of GSH levels is not reasonable because of its degradation in the plasma and poor cellular uptake [5–7]. GSH and its oxidized disulfide form (GSSG) are degraded by *γ*-glutamyltranspeptidase (GGT) via cleavage of the amino acid *γ*-glutamate from the N-terminal end of the peptide. GGT is located in the outer side of the cell membrane, and one of its functions, in cooperation with dipeptidases, is to provide cells with precursor amino acids needed for GSH *de novo* synthesis. To overcome the problems with GSH administration, several GSH analogues have been created to increase the GSH level and support the functionality of the GSH system [8]. We have previously designed and synthesized a library of peptidic GSH analogues [9]. For this study, two of them, UPF1 (Tyr(Me)-*γ*-Glu-Cys-Gly) and UPF17 (Tyr(Me)-Glu-Cys-Gly), were selected. Both molecules have an *O*-methyl-L-tyrosine residue added to the N-terminus of GSH-like Glu-Cys-Gly sequence to increase the antioxidativity and hydrophobicity. Previously, different groups have shown that various low molecular weight antioxidants, including melatonin, carvedilol, and its metabolite SB 211475, carry a methoxy moiety in their aromatic structures [10, 11]. The only structural difference between the peptides used is that UPF17 contains *α*-glutamyl moiety while UPF1 has *γ*-glutamyl moiety similarly to GSH. This switch from *γ*- to *α*-glutamyl moiety improved hydroxyl radical scavenging ability of UPF17 by approximately 500-fold compared to UPF1 whereas UPF1 itself is about 60-fold better hydroxyl radical scavenger than GSH [9]. In addition to being an excellent *in vitro* free radical scavenger, UPF1 has shown protective properties against oxidative damage in a global brain ischemia/reperfusion model and in an ischemia/reperfusion model on an isolated heart of Wistar rats [12, 13]. UPF1 and UPF17 have been shown to be nontoxic for K562 cells up to concentration of 200 *μ*M and UPF1 has no toxic effect on the primary culture of cerebellar granule cells at concentrations up to 100 *μ*M [9, 13].

Superoxide dismutases (SOD, EC 1.15.1.1.) are metalloproteins and the primary enzymes that keep cellular free radical production under control [14]. Cytosolic CuZnSOD is a homodimer (151 amino acids) with a molecular weight of 32500 Da and contains two cysteines (Cys57, Cys148) bound into an intramolecular disulfide bond and two free cysteines (Cys6, Cys111) [15, 16]. SOD catalyses the dismutation of superoxide into oxygen and hydrogen peroxide. Hydrogen peroxide as a diffusible cell damaging agent is further eliminated by glutathione peroxidase or catalase. One of the essential requirements for the biological activity of the glutathione peroxidase is glutathione as a cosubstrate. Consequently, SOD works synergistically with the glutathione against free radical damage.

This study examined the influence of UPF1 and UPF17 on CuZnSOD activity and intracellular GSH level in K562 cells. The aim of studying these tetrapeptides was to get information about whether and how the replacement of *γ*-peptide bond with *α*-peptide bond in the structure affects the bioactivities of the peptides. Additionally, we measured the stability of the peptides towards GGT to clarify their status in biologicalsystems and the pK_a_ values for thiol group dissociation.

## 2. Materials and Methods

### 2.1. Peptide Synthesis

UPF peptides were synthesized manually by solid phase peptide synthesis using Fmoc-chemistry and by machine using *tert*-Boc-chemistry as described previously [9, 17]. The purity of the peptides was >99% as demonstrated by HPLC on an analytical Nucleosil 120-3 C18 reversed-phase column (0.4 cm × 10 cm) and the peptides were identified by MALDI-TOF (matrix-assisted laser desorption ionization time-of-flight) mass-spectrometry (Voyager DE Pro, Applied Biosystems).

### 2.2. CuZnSOD Activity in K562 Cells

 The K562 cells (human erythroleukemia cells, obtained from DSMZ, Germany) were grown in T75 cell culture flasks in RPMI 1640 supplemented with 2 mM glutamine (PAA, Austria), 7.5% fetal calf serum, streptomycin (100 *μ*g/mL), and penicillin (100 U/mL) (all from Invitrogen, USA) at 37°C in a humidified 5% carbon dioxide atmosphere. Cells were seeded at concentration of 1.0 × 10^6^ per mL. Experiments were conducted 24 h after passage. Peptides (GSH, *α*-GSH, UPF1 and UPF17) diluted in DPBS (PAA, Austria) were added to the flasks containing the K562 cells. The cells were incubated with DPBS as control (Co) or with the peptide solution in a concentration range from 0.5 to 10 *μ*M for 24 h at 37°C. The peptide concentrations were chosen based on the GSH concentration in the blood plasma. After treatment, the cells were washed twice with DPBS and then lysed in water by keeping on ice for 20 min. Samples were centrifuged (12000 g) for 10 min and supernatants were transferred for experiments. The protein concentrations in the supernatants were determined by Lowry's method [18]. CuZnSOD activity was measured with the commercially available kit (Randox Laboratories Ltd, UK). This method employs xanthine and xanthine oxidase to generate superoxide radicals, which react with 2-(4-iodophenyl)-3-(4-nitrophenol)-5-phenyltetrazolium chloride to form a red formazan dye. The superoxide dismutase activity is then measured by the degree of inhibition of this reaction. One unit of SOD inhibited 50% of the rate of reaction.

### 2.3. Measurement of Total Glutathione

Concentrations of total glutathione (tGSH) were assessed by an enzymatic method of Tietze [19]. The homogenate was deproteinated by 10% solution of metaphosphoric acid (Sigma-Aldrich, Germany) in water and centrifuged at 12000 g for 10 min. The enzymatic reaction was initiated by the addition of NADPH, glutathione reductase, and 5,5′-dithio-*bis*-2-nitrobenzoic acid in buffer containing EDTA (Sigma-Aldrich, Germany). The change in optical density was measured after 15 min at 412 nm spectrophotometrically (Sunrise Tecan). Glutathione content was calculated on the basis of a standard curve.

### 2.4. Stability towards *γ*-Glutamyltranspeptidase

 1 mM UPF1 was incubated with 0.3 mg/mL equine kidney *γ*-glutamyltranspeptidase in 0.1 M Tris-HCl buffer pH 7.4, supplemented with 0.1% EDTA (Sigma-Aldrich, Germany) at 37°C for 1 h. 6 mM Gly-Gly was added as an acceptor for *γ*-Glu moiety [20]. GSH was incubated with GGT under the same conditions as the control. The samples were heat-inactivated, centrifuged at 10000 g and +4°C for 5 min, and kept on ice until analyzed. Supernatants were analyzed on a Prominence HPLC (Shimadzu, Japan) and Q-Trap 3200 (Applied Biosystems, USA) mass spectrometry tandem. Luna C18 100 × 2 mm, 3 *μ*m column from Phenomenex was used for sample separation. Solvent A was a mixture of 99.9% water and 0.1% HCOOH, and solvent B was a mixture of 99.9% acetonitrile and 0.1% HCOOH (mass spectrometry grade, Riedel-de Haėn, Germany). Samples were eluated at a flow rate of 0.1 mL/min, gradient started with 5 min at isocratic flow of solvent A, concentration of solvent B increased up to 30% in 25 min, followed by wash with 100% solvent B in 20 min. Enhanced MS scans were performed in negative mode with rate 1000 amu/s between mass range 50–1700 Da. Ionspray voltage was set to −4500 V, declustering potential to −30 V and entrance potential to −10 V.

### 2.5. pK_a_ of Thiol Groups

The ratio of thiol and thiolate concentrations were measured spectrophotometrically at 240 nm on a PerkinElmer Lambda 25 spectrometer similarly as previously for GSH and *α*-GSH [21]. 1 mL of 50 *μ*M peptide solution in phosphate buffered saline (Calbiochem, USA) was titrated with 5 *μ*L volumes of 1 M NaOH and pH and absorbance changes were determined after each addition. The results were corrected to consider the dilution of the assay mixture during titration.

### 2.6. Statistical Analysis

Data were analyzed using GraphPad Prism version 4.00 for Windows (GraphPad Software, San Diego, CA, USA). The results on the graphs are presented as the mean ± standard error of the mean (SEM).

## 3. Results

### 3.1. CuZnSOD Activity

**Figure 1:**
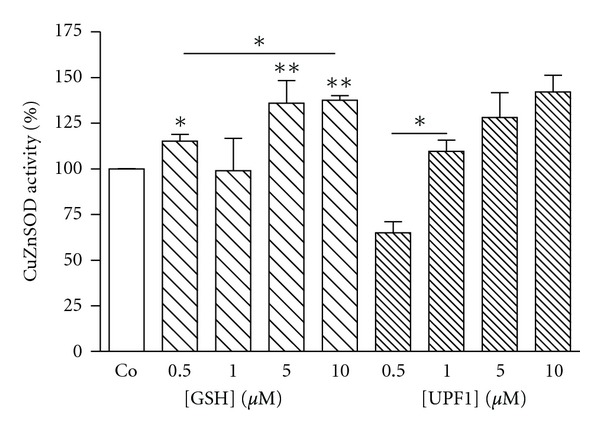
Modulation of CuZnSOD activity by GSH and UPF1 in K562 cells. The CuZnSOD activity of Co is 100%. *P < 0.05; **P < 0.01, GSH and UPF1 versus Co; *n* = 4–8.

**Figure 2:**
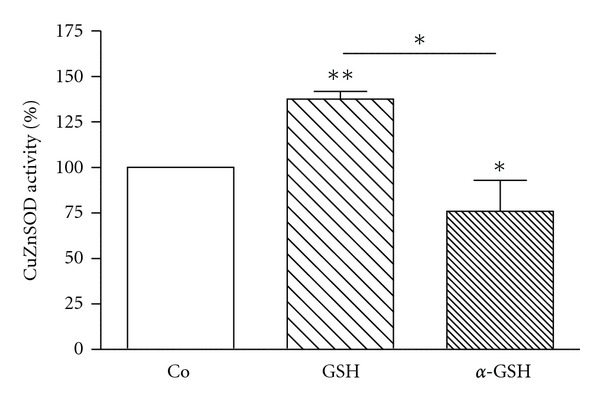
Modulation of CuZnSOD activity by GSH and *α*-GSH (10 *μ*M) in K562 cells. The CuZnSOD activity of Co is 100%. *P < 0.05; **P < 0.01, 10 *μ*M GSH or *α*-GSH versus Co; *n* = 4–8.

**Figure 3:**
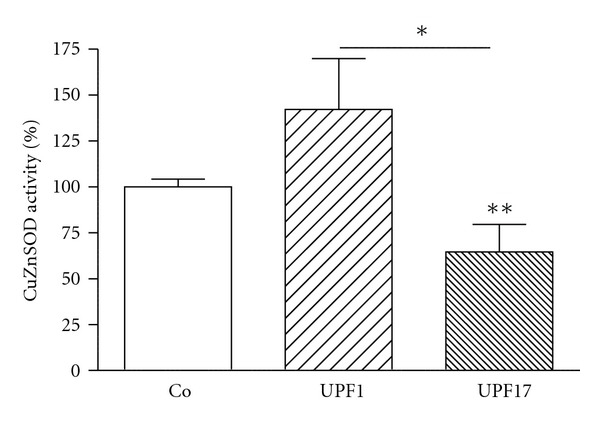
Modulation of CuZnSOD activity by UPF1 and UPF17 (10 *μ*M) in K562 cells. The CuZnSOD activity of Co is 100%. *P < 0.05; **P < 0.01, 10 *μ*M UPF1 or UPF17 versus Co; *n* = 4–8.

K562 cells were incubated with investigated peptides (GSH, *α*-GSH, UPF1, and UPF17) for 24 h at four different concentrations: 0.5, 1.0, 5.0, and 10 *μ*M. GSH showed a concentration-dependent activating effect on CuZnSOD activity, whereas 10 *μ*M GSH increased the enzyme activity by 38% (Figure 1). *α*-GSH had an inhibiting effect (24%) on the enzyme activity but only at the highest concentration used (10 *μ*M) (Figure 2). UPF1 increased the activity of CuZnSOD at concentrations of 1.0, 5.0, and 10 *μ*M, but at concentration of 0.5 *μ*M showed an inhibition of the enzyme activity (Figure 1). The activation rate was concentration dependent. Contrary to UPF1, UPF17 showed an inhibitory effect and the inhibition was not concentration dependent. UPF1 increased and UPF17 decreased the activity of CuZnSOD at peptide concentration of 10 *μ*M by 42% and 35%, respectively (Figure 3). As the peptide concentration of 10 *μ*M was the most effective, it selected for the comparison.

### 3.2. Intracellular GSH Level

**Figure 4:**
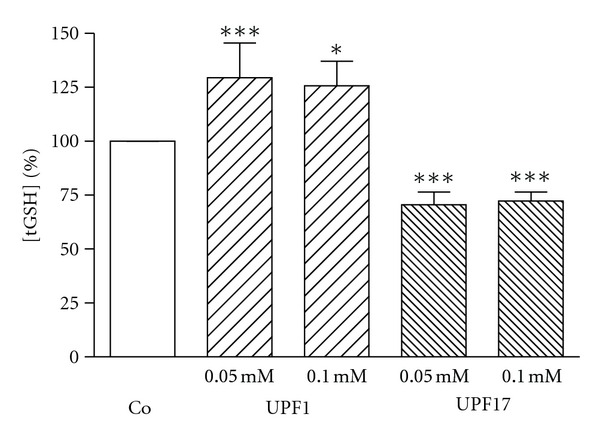
Alteration of tGSH concentration by UPF1 and UPF17 in K562 cells. The tGSH concentration of Co is 100%. *P < 0.05, ***P < 0.005, UPF1 or UPF17 versus Co; *n* = 6–8.

K562 cells were incubated with UPF1 and UPF17 peptides for 3 h at concentrations of 0.05, 0.10, and 0.5 mM. Previous experiments have shown that at these concentrations UPF peptides are effective free radical scavengers and are biologically active. In addition, the 0.5 mM concentration was chosen to match with millimolar GSH concentration in number of cells. UPF1 increased and UPF17 decreased GSH concentration at concentrations of 0.05 and 0.1 mM by 29% and 26% or 26% and 28%, respectively (Figure 4). No statistical difference in tGSH concentration compared to control after incubation with 0.5 mM peptides, the highest concentration used, was detected.

### 3.3. Degradation by GGT

After incubating GSH with GGT, GSH was degraded and *γ*-Glu moiety was transferred to an acceptor Gly-Gly dipeptide, resulting in a new compound in mass spectra with MW 261.2 Da [*γ*-Glu-Gly-Gly]. The question arose: can the bond between *γ*-glutamate and cysteine be degraded by GGT in UPF1, where the access to the bond is obstructed by an additional amino acid methylated tyrosine? Results obtained from the mass spectrometry measurements demonstrated that UPF1 is not degraded by GGT as the expected peaks with or without acceptor dipeptide MW 438.4 Da [Tyr(Me)-*γ*-Glu-Gly-Gly] or 324.3 Da [Tyr(Me)-*γ*-Glu], respectively, did not appear. During the incubation, UPF1 was dimerised over disulphide bridge. GGT is also able to breakdown dimeric form of GSH, but degradation of dimerised UPF1 was not detected.

### 3.4. pK_a_

The pK_a_ values of thiol groups of the peptides were measured. For GSH and *α*-GSH, the values were 9.0 ± 0.3 and 9.1 ± 0.1, respectively, whereas pK_a_ values for UPF peptides were slightly higher: 9.3 ± 0.1 for UPF1 and 9.4 ± 0.2 for UPF17.

## 4. Discussion

The present study focused on the effects of UPF1 and UPF17 on CuZnSOD activity and intracellular GSH level in K562 cells. For the first time we described and compared counterpoint biological activities of structural antioxidative peptide analogs differing from each other by spacial arrangement of Glu residue (*γ*-peptide bond in UPF1 changed to the *α*-peptide bond in UPF17). Previously we have shown that UPF1 and UPF17 have a tendency for MnSOD activation. However, the *γ*-glutamyl moiety containing UPF1 needed more time for MnSOD activation compared to UPF17, which had the effect already after 5 min incubation. UPF1 and UPF17 have also different influence on glutathione peroxidase activity (GPx): at higher concentrations than used in *in vivo *experiments, both UPF1 and UPF17 inhibited activity concentration dependently whereas the *α*-peptide bond containing UPF17 had stronger inhibitory effect [22]. In the present work we investigated how the replacement of *γ*-peptide bond with *α*-peptide bond on GSH and its analogue UPF1 affects CuZnSOD activity and level of GSH in K562 cells. The results showed that *γ*-Glu moiety containing GSH and UPF1 stimulated CuZnSOD activity and increased intracellular tGSH level, whereas *α*-GSH and UPF17, which have *α*-Glu moiety in the structure, inhibited enzymatic activity and decreased GSH level. The stability of UPF1 towards GGT activity indicated that UPF1 affects GSH level and CuZnSOD activity as intact molecule instead of being a GSH precursor. Previously, it has been shown that GSH and UPF1 are able to act as signaling molecules through G-protein activation in frontocortical membrane preparations [23]. It has been reported that plasma membranes have specific binding sites of GSH which have an interaction with the glutamate binding sites [24]. By this way GSH and UPF peptides may affect the metabolism of cells as signal molecules. The effects on the level of GSH and CuZnSOD activity may be different depending on the replacement of *γ*-peptide bond with *α*-peptide bond. GSH has been shown to bind to ionotropic glutamate receptors via gamma-glutamyl residue in the nervous tissue [25]. Additionally, glutamate receptors have been found also in the plasma membrane of megakaryocytes and rat erythrocytes [26, 27]. By interacting with the latter receptors, GSH and UPF peptides may affect the metabolism of cells as signal molecules through the PKC pathway and affect CuZnSOD activity. The various effects of the studied molecules may be caused by structural differences between the GSH and UPF peptides (replacement of *γ*-peptide bond with *α*-peptide bond).

UPF1 and UPF17 have also shown different effects in free radical scavenging experiments. According to the classification of kinetic behavior by Sánches-Moreno et al., UPF17 is classified as fast and UPF1 as intermediate DPPH radical scavenger [9, 28]. *In silica *modeling of noncovalent complex formation by docking calculations revealed a more affine complex between DPPH radical and *α*-GSH compared to the complex with GSH [21]. This raised a question about pK_a_ values for the thiol groups of UPF peptides. Previously, the change of *γ*-peptide bond to *α*-peptide bond has also been investigated for GSH and its *α*-analogue: pK_a_ of thiol groups were similar for GSH and *α*-GSH (9.0 ± 0.1 and 9.1 ± 0.1) [21]. The comparison of these results with current measurements for UPF1 and UPF17 demonstrated that pK_a_ value is rather influenced by the addition of a methylated tyrosine moiety to the GSH backbone than by the change of the peptide bond type. Smaller pK_a_ values for GSH and its *α*-analogue showed that these molecules donate the sulfhydryl proton more easily than UPF peptides; however, UPF peptides are better radical scavengers. This indicates that reactive species elimination does not depend only of the reactivity of the thiol group.

 The results of the current paper show that *γ*-peptide bond and *α*-peptide bond containing UPF peptides may influence enzyme activities in different direction, which offers a wider perspective for the usage of glutathione analogues as protective diverse regulators of the oxidative state.
